# The National Care of the Sick and Wounded

**Published:** 1915-04-17

**Authors:** 


### The Hospital

The Workers' Newspaper of Administrative Medicine and Institutional Life, Administration, National Insurance and Health.

No. 1504, Vol. LVIII SATURDAY, APRIL 17, 1915. PRICE ONE PENNY

### THE NATIONAL CARE OF THE SICK AND WOUNDED.

Thanks to the cordial and loyal co-operation of our esteemed contributors, we are able in this week's issue to offer to our readers a graphic account of the organisations and arrangements by which sick and wounded soldiers are conveyed from the fighting line to the shelter and safety of British hospitals. There seemed to be a special claim on a journal devoted in the widest sense to the efficient ordering of the machinery necessary for the care of the sick to endeavour to provide a representation of the working of this machinery under the stress of the present crisis. It is this claim which we have endeavoured to satisfy. For obvious reasons the task has not been unattended with difficulties. Many of those who are most intimately concerned with the control and regulation of the Transport Service are so overwhelmed with the demands of active service that, however great their good will, descriptive and educational exercises are for the present beyond their power. To others, for large and public reasons which we all respect, the pen is in the meantime forbidden. Fortunately, we have been able to secure efficient help from recognised and authoritative sources; and we have the assurance that our purpose commands the sympathy of those who are set in highly responsible places. Alike for active co-operation and for privately expressed encouragement, we have the pleasure here to record our sincere gratitude; and we do not doubt that our readers will wish to associate themselves heartily with this sentiment.

The war in its meaning and purpose is, of course, a matter of high national concern, and on this ground alone there is a legitimate and widespread desire for an assurance that those who risk life and limb in the nation's cause will receive adequate care and attention should illness or wounds strike them to the ground. The circumstances of the hour combine to render this interest more acute and to lend to it an intimate and personal application. Few are the homes of our land from which some member has not gone to take his risk in the great struggle; and many to whom the Army was formerly little more than a picturesque display are now anxiously concerned with its care and welfare. The law of sacrifice has made its demand on the widest scale, and those who have given husbands and sons and friends—dearer often than life itself—follow with solicitude not only the general fortunes of the great campaign but also its personal meaning. It is common knowledge that the immediate future is to produce events which will heighten and sharpen these feelings, and that news from the Front will to increasing numbers mean not merely the cause of the nation but also, and with not less concern, the fate of individuals. General assurances cannot satisfy this legitimate apprehension. Something more real and precise is needed, and this we have endeavoured to supply in the hope that detailed descriptions will have at least the measure of comfort conveyed in the knowledge that no skill or care shall be wanting for brave men wounded and suffering in the nation's interest.

Another aspect of the subject with which our present issue is mainly occupied may be described as educational. The complexities of modern armies are probably incapable of full realisation except to the initiated and to those who are charged with technical duties. This is true more particularly of the Services whose work is conducted behind the scenes and out of the public eye. Quite naturally the activities of the combatant ranks and the shock of battle engage the general attention, whilst the agencies which minister to these and make them possible have a less dramatic quality and are largely conducted in secrecy, or at least out of view. None the less these subsidiary agencies are essential contributions to the great end, and without their efficiency the welfare and success of the fighting forces are impossible. Equally this efficiency requires foresight, personal effort, and the ability to anticipate and to meet emergencies. It is to be remembered, too, that the call here is made apart from the stimulating and exciting influences of conflict. On the contrary, some of the best work is done in obscurity and in circumstances which demand self-effacement without any hope of recognition or reward. It is well to honour our soldiers who bravely acquit themselves amidst the risks of the battlefield, but it is well also to remember those not less heroic souls who achieve duty in its duller shapes. Some of these are doubtless hidden from public view even more than those who are charged with the care of the sick and wounded, but a recital of the activities of one group of non-combatants may help towards an appreciation of the labours of others who work in silence for the common cause.

We cultivate yet another hope from our Special Issue. For many years this Journal has advocated the care of the sick as a matter of national and personal concern. Our voluntary hospital system stands as a recognition of the justness of this principle, and the war entitles us to hope that they will not be wholly forgotten. Even to our wounded soldiers our voluntary hospitals have proved to be essential. Not less necessary are they to the State and to the people in days when peace and not war is the national concern. It is surely not unreasonable to hope that the display in conspicuous fashion of the organisations necessary for the care of the wounded in battle, will quicken interest in the institutions which on similar lines discharge a national obligation in more peaceful times. Directly or indirectly everyone is desirous of helping the first of these. We suggest that the claims of patriotism extend equally to the hospitals.

